# Effect of relative density on dynamic mechanical behavior and deformation mechanisms of porous titanium under coupled high-temperature and high-strain-rate conditions

**DOI:** 10.1080/14686996.2025.2580925

**Published:** 2025-10-28

**Authors:** Dong Yang, Mingyu Li

**Affiliations:** Department of Mechanical Engineering, Anhui University, Hefei, China

**Keywords:** Porous titanium, relative density, dynamic response, high-temperature, high-strain-rate

## Abstract

The influence of relative density on the dynamic mechanical behavior of porous titanium under combined high-temperature and high-strain-rate conditions is investigated. Using validated finite element models based on three-dimensional Voronoi tessellations, simulations of Split Hopkinson Pressure Bar (SHPB) tests were conducted across a range of relative densities (0.3–0.6), strain rates (3000–8000 s^−1^), and temperatures (25–550 °C). Results demonstrate that increasing relative density from 0.3 to 0.6 increases the yield stress by 511.8%, attributed to enhanced cell-wall interactions and a concomitant shift in deformation mechanisms. Strain rate strengthening and thermal softening compete, with high relative density amplifying both effects. The stress-strain curves exhibit three characteristic regimes: linear elasticity, plateau, and densification, where higher relative density shortens the plateau stage and advances densification onset. Low-density specimens (*ρ*_*r*_ < 0.5) undergo layer-by-layer collapse dominated by cell-wall bending, while high-density specimens (*ρ*_*r*_ > 0.5) exhibit matrix-dominated triaxial compression with reduced localized deformation. Quantitative analysis of regionally partitioned displacement confirms that strain rate intensifies the magnitude of localized deformation, whereas temperature primarily induces global softening. These insights provide a predictive framework for designing porous titanium architectures with tailored dynamic performance in extreme environments.

## IMPACT STATEMENT

The dynamic mechanical behavior of porous titanium under combined high-temperature and high-strain-rate conditions, governed by relative density, is elucidated, and its critical implications for designing tailored porous architectures in extreme dynamic environments are highlighted.

## GRAPHICAL ABSTRACT

**Figure:**
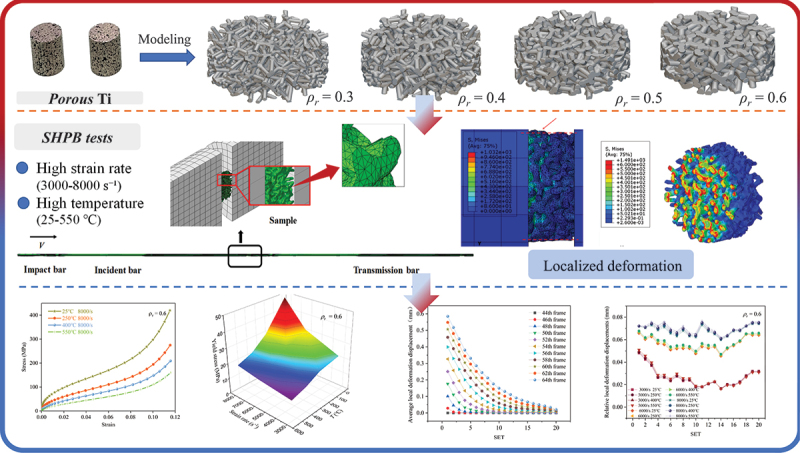

## 1. Introduction

With the surging demand for lightweight impact-resistant materials in aerospace, defense, and related industries, porous metallic materials have emerged as a research focus due to their exceptional mechanical properties: combining low density, high specific strength, and superior energy absorption capacity [1]. Among artificial porous metallic materials, porous titanium has found extensive applications in load-bearing sandwich core structures, aircraft heat dissipation/insulation layers, heat exchangers, and catalyst substrates [2]. However, the extreme service conditions in these applications impose stringent demands on integrated structural integrity and functional performance. Consequently, elucidating the relationship between porous architecture and the underlying dynamic deformation mechanisms under such extreme thermo-mechanical conditions is critical for material design and application.

Regarding the mechanical response of metallic foams under extreme thermo-mechanical loading, strain rate and temperature exhibit distinct yet competing effects. Zhang et al. [3] demonstrated that titanium foams with 45–55% porosity exhibit significant strain rate strengthening under high strain rates (1000–4000 s^−1^), where dislocation strengthening dominates the enhanced impact resistance by inhibiting plastic deformation. Alvandi-Tabrizi et al. [4] reported that composite metal foams show elevated yield and plateau strengths at high strain rates, attributed to the strain rate sensitivity of the parent matrix and pressurization of entrapped air within closed cells, which restricts cell wall buckling. Siegkas et al. [5] also noted that titanium foams display higher yield stress and strain hardening rate with increasing strain rate, especially for those with lower relative density, as micro-inertial effects in pore structures amplify the strain rate response. Khezrzadeh et al. [6] found that A356 aluminum foams exhibit improved strength and energy absorption at cryogenic temperature (−196℃) due to brittle deformation of cell walls, while high temperature (250℃) induces matrix softening and a transition to ductile deformation, reducing both strength and stress fluctuations in the plateau region. Lu et al. [7] reported similar softening behavior for magnesium alloy foams, where compressive strength and energy absorption capacity decrease monotonically with temperature (25–500℃), accompanied by a shift in failure mechanism from brittle shear fracture to a combination of brittle fracture and ductile cell wall bending. Liang et al. [8] further observed that the compressive strength of AlSi alloy foam increases with strain rate across temperatures (25–560℃), with the dynamic increase factor rising from 1.81 at 25°C to 3.50 at 560°C, indicating that strain rate-induced strengthening becomes more prominent as temperature increases. Wang et al. [9] emphasized that aluminum foam at high temperatures (up to 773K) shows more pronounced strain rate sensitivity but reduced stress drop coefficients, as plastic bending of cell walls replaces buckling and tearing as the dominant deformation mode, weakening the temperature-induced softening effect to some extent. Collectively, these studies highlight the competitive interplay between strain rate and temperature, with the balance of these effects governing the overall thermo-mechanical response of metallic foams in extreme service conditions. However, a systematic quantification of how this competitive interplay is fundamentally governed by the material’s morphological factors remain less explored, particularly for porous titanium under coupled extreme conditions.

The performance of porous materials is strongly governed by their morphological factors including pore size, pore distribution, porosity, relative density, and density distribution, etc. Cui et al. [10] investigated the effect of porosity on dynamic response of additive manufacturing Ti-6Al-4 V alloys and found porosity significantly affects yield and spall behaviors. Hugoniot elastic limit and spall strength decrease monotonically with increasing porosity, and voids collapse under shock compression to cause spallation in highly porous. Zhao et al. [11] investigated the mechanical behavior of powder-sintered porous titanium with different pore sizes (mean: 30 μm and 120 μm) via high strain rate (up to 6600 s^−1^) and quasi-static compression tests. The yield stress exhibited two stages of strain-rate sensitivity with a transition at 1600 s^−1^, with the sensitivity in the second stage being an order of magnitude higher. At the same strain rate, both yield stress and strain field homogeneity decreased significantly as pore size increased. Li et al. [12] studied the mechanical properties of porous Ti with 200–400 μm pores, finding that elastic modulus and yield stress declined considerably with increasing pore size. However, Tuncer et al. [13] reported the opposite trend for porous Ti with pore sizes ranging from 200 μm to several millimeters: in their study, elastic modulus and yield stress increased linearly with larger pore sizes. Thus, the response of material mechanical properties to their structure still needs to be analyzed in combination with other structural parameters.

Relative density, defined as the ratio of the density of the porous material to the density of its solid constituent, differs fundamentally from porosity (volume fraction of pores) in that it directly reflects the volume fraction of solid material, making it a more critical parameter for mechanical performance characterization [14]. Based on the micromechanical model by Gibson and Ashby [15], the material properties of metallic foams can be quantitatively described through power-law dependencies on both the properties of the base material and the relative density. Saleem et al. [16] revealed through quasi-static and dynamic experiments that the microstructure significantly influences the compressive behavior of open-cell nickel foam (OCNF-A), with its plateau stress increasing proportionally to relative density. Michailidis et al. [17] employed X-ray tomography to analyze strut deformation and buckling phenomena during the deformation of open-cell nickel foam, while evaluating the impact of relative density on its mechanical response. SEM micrographs illustrating cross-sectional deformation of strut walls in open-cell foam under dynamic loading. Mondal et al. [18] investigated the compressive behavior of aluminum foam with relative densities ranging from 0.08 to 0.13, demonstrating that plateau stress increases with relative density but remains strain-rate insensitive. Through quasi-static experiments, Muchhala et al. [19] observed that cells in aluminum composite foam initially undergo bending (stretching), followed by shear and crushing. The resulting fine fragments promote sample densification, with the material’s densification strain exhibiting a linear relationship with relative density. Fu et al. [20] summarized the correlation between relative density and yield stress from existing literature. Yang et al. [21] reported over a tenfold increase in plateau stress when the relative density of aluminum foam rises from 0.08 to 0.36, highlighting that adjusting relative density enables tailored porous materials for diverse applications. Kádár et al. [22] investigated the indentation behavior of Alporas foams of different relative densities, and found that the plastic Poisson’s ratio increases with rising relative density. The magnitude of relative density exerts a pronounced influence on the mechanical behavior of porous metals, particularly under dynamic loading conditions. However, prior studies lack a systematic investigation quantifying the coupled effects of relative density, temperature, and strain rate on dynamic compressive response, particularly for porous titanium under extreme thermo-mechanical conditions.

This study employs a combined experimental and computational approach to investigate the dynamic response of porous titanium. Split Hopkinson Pressure Bar (SHPB) tests and corresponding finite element models, validated against the experimental data, are utilized. The validated models enable a detailed analysis of the effects of relative density on stress-strain response, rate-temperature coupling effects, and deformation mechanisms under coupled high-temperature and high-strain-rate conditions. The findings provide a predictive framework for designing porous titanium architectures with tailored dynamic performance.

## 2. Materials and methods

### 2.1. Porous titanium

**Figure 1.**
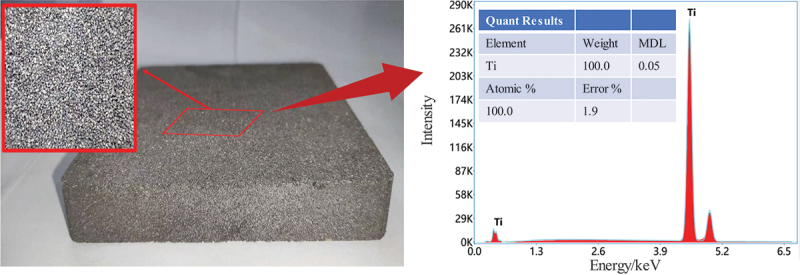
Porous titanium and its EDS spectrum.

The porous titanium specimens employed in this study were prepared via powder metallurgy techniques. Titanium powder was subjected to sintering in a vacuum atmosphere (with a pressure of 6 × 10^−3^ Pa) at 980°C, after which it was slowly cooled down to ambient temperature inside the furnace. Variations in relative density can be achieved by regulating the particle size of the titanium powder, the compaction pressure applied, or the duration of the sintering process. Figure 1 shows porous titanium with a relative density of 0.59, along with its energy-dispersive X-ray spectroscopy (EDS) spectrum. The matrix, composed exclusively of Ti, indicates that the material is Grade 1 commercially pure titanium (TA1), which complies with the ASTM B265 standard.

### 2.2. SHPB experimental setup

**Figure 2.**
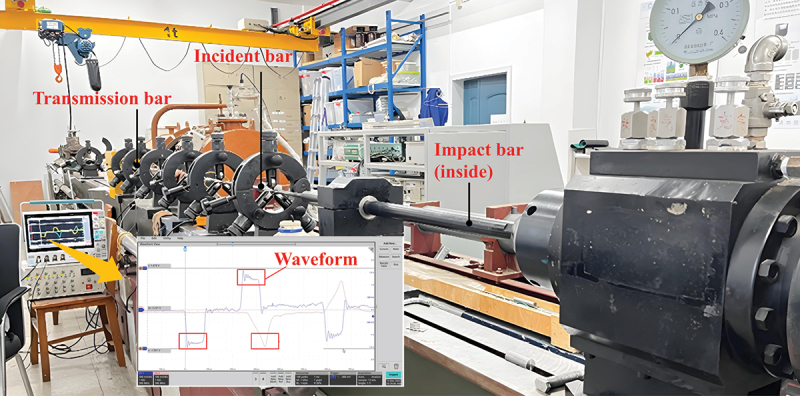
Schematic diagram of the SHPB experimental system.

Dynamic loading experiments were conducted on the Split Hopkinson Pressure Bar experimental system (Di Mei Tai Precision Technology Co., Ltd., China). The Split Hopkinson Pressure Bar system is illustrated in Figure 2. It primarily comprises the main body of the pressure bars and a data acquisition system. The main body of the pressure bars consists of the impact bar, incident bar, and transmission bar, with the specimen positioned between the incident bar and transmission bar. The impact specimen is a cylindrical specimen with dimensions of Ф4 × 2 mm. The parameters of the impact bar, incident bar, and transmission bar are presented in Table 1.

**Table 1.** Geometric parameters of the SHPB bars.

Size (mm)	Impact bar	Incident bar	Transmission bar
Diameter	12	12	12
Length	200	1500	1500

During the experiment, the end faces of the specimen are first coated with Vaseline and then clamped between the incident bar and the transmission bar. The impact bar is propelled by compressed gas to impact the incident bar, generating a rectangular pressure pulse that propagates forward in the incident bar. This pulse is defined as the incident wave. Voltage signals during wave propagation are recorded by a pair of resistance strain gauges attached to the mid-section of the incident bar. These signals are subsequently converted by an ultra-dynamic strain gauge connected to a Wheatstone bridge, and finally, the waveform is displayed and recorded on an oscilloscope. When the pressure pulse reaches the front end of the specimen, a portion of the wave is absorbed by the specimen, while the remaining portion is reflected along the incident bar and captured by the strain gauges. This reflection occurs at the contact interface between the specimen and the incident bar. The pressure wave that penetrates the specimen continues to propagate forward and ultimately reaches the rear end of the specimen. At the rear end, part of the pressure wave is transmitted through the specimen into the transmission bar, forming a transmitted wave. In high-temperature SHPB experiments, the temperature of the heating furnace is first set. After the temperature is raised to the preset value, a 10-minute soaking period is maintained using a timer to ensure uniform temperature distribution within the specimen. At the conclusion of the soaking period, the impact bar is launched to conduct the experiment.

### 2.3. Voronoi based finite element modeling

**Figure 3.**
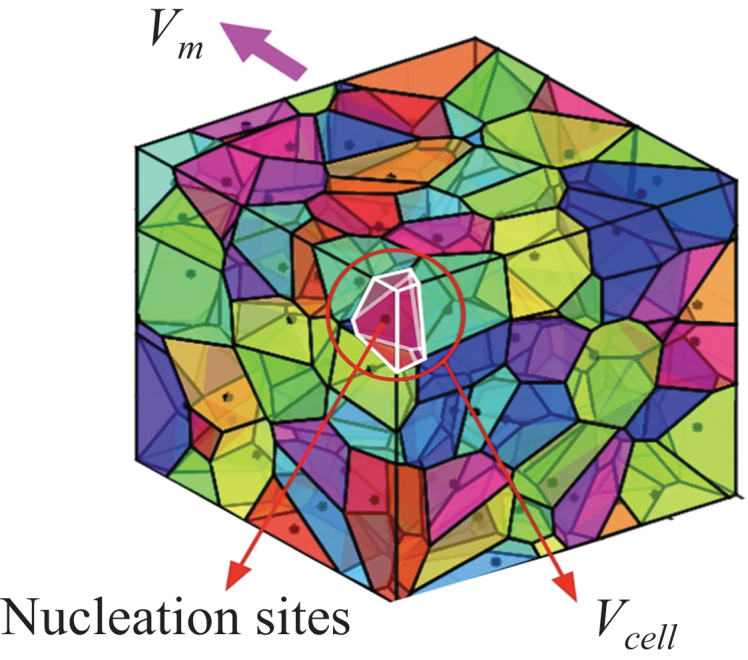
Schematic of 3D Voronoi geometric model.

To characterize the structural features of porous titanium, the geometric model of porous titanium was constructed using a three-dimensional random Voronoi tessellation technique, which can well characterize the degree of randomness and distribution of cell pores in foam metals [23,24] Figure 3. schematic diagram illustrating the Voronoi-based modeling concepts [25]. The basic principle is that within a region of specified volume *V*_*m*_, *N* uniformly distributed nucleation sites are randomly introduced. The inter-site distances are constrained to satisfy a specific condition: the distance between any two sites must be at least a predefined value *δ*, defined as:

(1) $$\delta_{\min}=(1-k)\delta_{0}$$

where, *k* denotes the irregularity of the porous metal, characterizing the mesostructural irregularities. *δ*_0_ represents the minimum inter-nucleation-site distance in a regular tetrakaidecahedron model, given by:

(2) $$\delta_{0}=\sqrt{3}(V_{\mathrm{cell}}/4{)}^{1/3}$$

where *V*_*cell*_ is the volume of a single cell in the regular tetrakaidecahedron.

The generation of the porous titanium Voronoi model employed in this study entails three sequential steps:(1) Random generation of nucleation sites: in three-dimensional space, *N* nucleation sites are generated via a stochastic algorithm within a region whose volume matches that of the actual sample, strictly conforming to the constraint specified in Equation (1). This procedure ensures that the distribution of nucleation sites is both random and subject to distance constraints, thereby accurately simulating the spatial arrangement of cell nucleation sites in real porous metals. (2) Construction of the Voronoi sructure: Building upon the successfully generated nucleation sites, the skeletal framework of the model is incrementally constructed by connecting these sites, following which a complete Voronoi structure is formed. Importantly, this process necessitates precise calculation of inter-nucleation-site distance relationships between each site and its surrounding neighbors. (3) Generation and optimization of cell wall (strut) structures: Leveraging the aforementioned Voronoi structure, cylindrical cell walls (struts) are generated using advanced modeling techniques. By finely tuning the minimum inter-nucleation-site distance and the thickness of the cell walls, the relative density of the model can be effectively regulated. This step is pivotal for simulating and quantitatively investigating the mechanical behavior of porous metals with varying relative densities.

**Figure 4.**
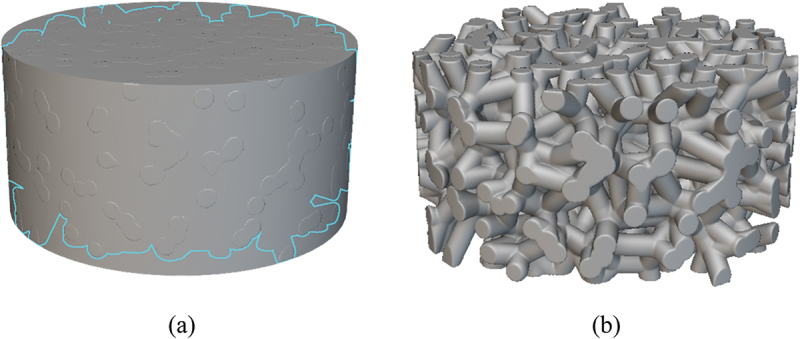
Geometrical models for relative density calculation: (a) Solid cylindrical reference model; (b) Porous titanium model with Voronoi structure.

In this article, cylindrical specimens with dimensions of Ф4 × 2 mm were constructed for subsequent dynamic loading experiments. The number of nucleation points in the Voronoi model was set as 3000, and the relative density of the porous titanium was controlled by adjusting the cell wall thickness. The relative density of each model was verified by calculating the volume ratio between the porous structure and a solid cylindrical model of identical dimensions within the computational software, as illustrated in Figure 4. The relative density (*ρ*_*r*_), defined as the ratio of the density of the porous material to the density of its solid constituent, was calculated as:

(3) $$\rho_{r}=\frac{V_{s}}{V_{0}}$$

where *V*_*0*_ represents the total volume of the material, *V*_*S*_ signifies the volume of the dense matrix material.

The porosity was determined using the following formula:

(4) $$\theta =\frac{V_{p}}{V_{0}}=\frac{V_{0}-V_{s}}{V_{0}},$$

where *θ* denotes the material porosity, *V*_*p*_ corresponds to the pore volume. The relationship between relative density *ρ*_*r*_ and porosity *θ* is defined as

(5) $$\theta =1-\rho_{r},$$

**Figure 5.**
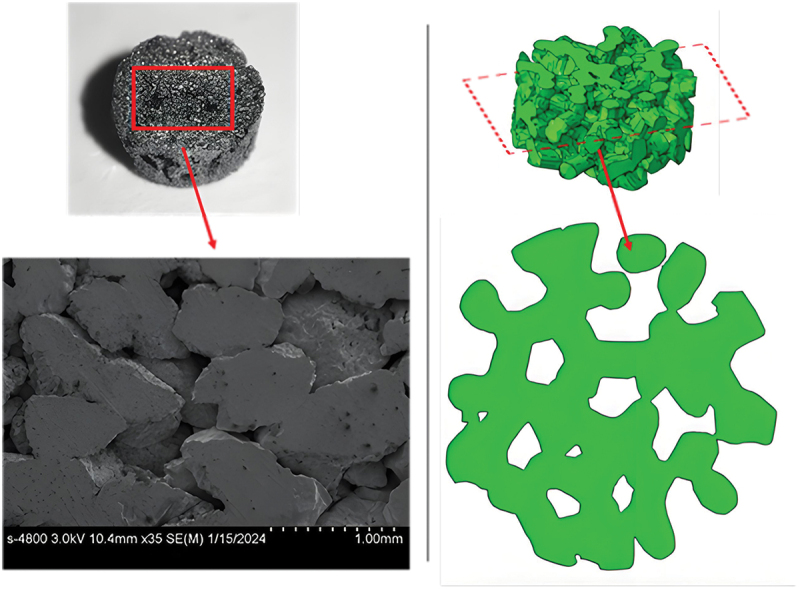
Comparison of regional structures between experimental samples and geometric models of porous titanium.

To verify the model accuracy, corresponding regions of equivalent size were selected between the experimental samples and the porous titanium geometric models for statistical analysis, as illustrated in Figure 5. Pore sizes were quantified using Image-Pro, with the diameter measured along the minor axis of the cell pores as the reference. The statistical parameters presented in Table 2 demonstrate that the experimental samples and Voronoi models exhibit structural consistency.

**Table 2.** Statistical table of structural parameters for porous titanium specimens and Voronoi models.

Data	Porosity	Maximum pore diameter (mm)	Average pore diameter (mm)	Minimum pore diameter (mm)	Standard deviation (σ)
Specimen	0.612	0.973	0.293	0.019	0.015
Voronoi model	0.608	0.961	0.338	0.012	0.021

**Figure 6.**
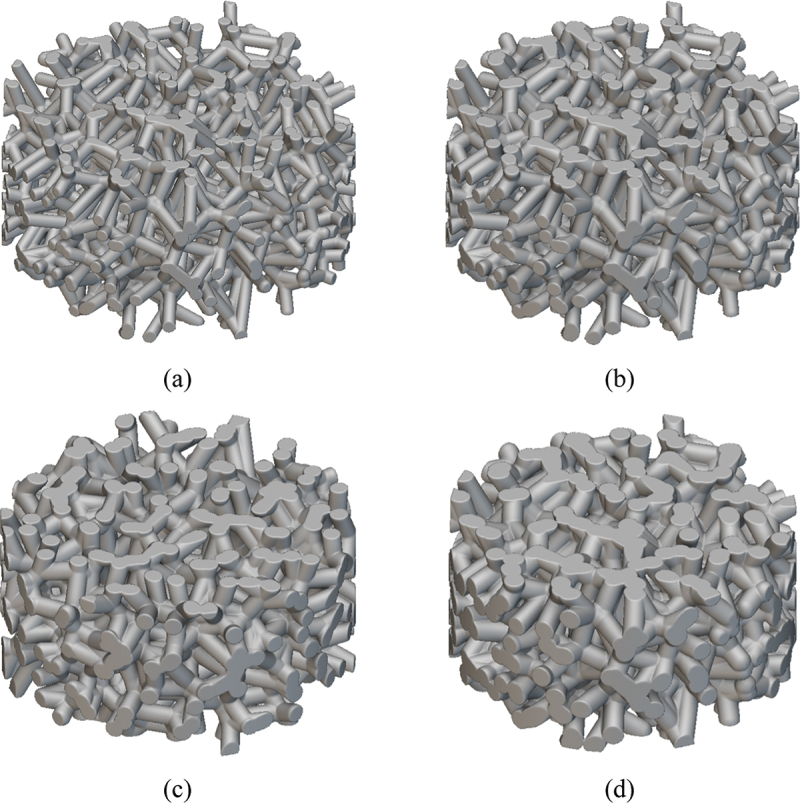
Voronoi-based finite element models of porous titanium with different relative densities: (a) *ρ*_*r*_ = 0.3, (b) *ρ*_*r*_ = 0.4, (c) *ρ*_*r*_ = 0.5 and (d) *ρ*_*r*_ = 0.6.

In the present study, four specimen models with distinct relative densities (0.3, 0.4, 0.5, and 0.6) were constructed, as depicted in Figure 6, to investigate the effect of the material’s relative density on its dynamic mechanical behavior.

### 2.4. Simulation setup and model validation

**Figure 7.**
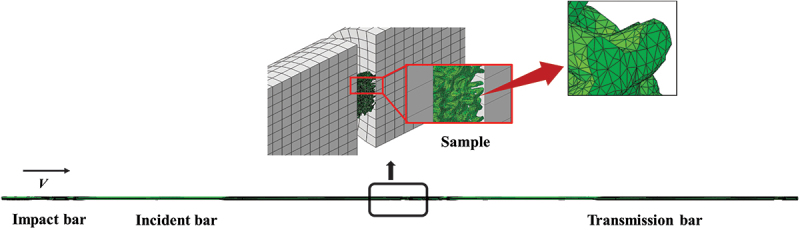
Finite element model of the SHPB setup for porous titanium compression.

Alongside the Split Hopkinson Pressure Bar (SHPB) experiments, a meso-scale finite element model of the SHPB system was established using the finite element software ABAQUS/Explicit. As a powerful tool for investigating the mechanical behavior of porous materials, this meso-scale model accurately captures the material’s microscopic deformation mechanisms and impact response characteristics. It is employed to characterize the dynamic mechanical responses of porous titanium with different relative densities over a wide range of strain rates and temperatures, and to observe the deformation behaviors of the material at various scales. In the numerical simulation, a rigid impact bar is employed for the impact. The impact bar is set with a Young’s modulus of 200 GPa, a Poisson’s ratio of 0.3, and a density of 7.85 g/cm^3^ [26]. The mechanical properties of the transmission bar and the impact bar are identical. Using general contact, the friction coefficient between the rigid plate and the specimen is 0.2. Different elevated temperatures were applied to the porous model within a predefined temperature field. The numerical model for dynamic compression test analysis of porous titanium is shown in Figure 7.

The porous titanium models were meshed with hybrid solid elements combining C3D10 (standard quadratic tetrahedron) and C3D10M (modified quadratic tetrahedron for explicit dynamics). A mesh sensitivity analysis was performed to validate the element selection. This analysis confirmed that further refinement beyond the chosen mesh density resulted in variations of less than 2% in key output parameters (e.g. stress concentration factors, global stress), ensuring sufficient calculation accuracy.

The Johnson-Cook (JC) model [27] was employed to simulate the constitutive behavior of the titanium matrix. The JC equation, as represented by Equation (6), integrates the influences of strain hardening, strain rate hardening, and temperature, with each influence considered independently from the others.

(6) $$\sigma =[A+B\varepsilon^{n}][1+C\ln\frac{\dot{\varepsilon }}{{\dot{\varepsilon }}_{0}}][1-T^{*m}]$$

where σ represents the flow stress, *A* denotes the quasi-static yield stress at reference strain rate and temperature, *B* represents the strain hardening modulus, *n* represents the strain hardening exponent, *C* represents the strain rate sensitivity coefficient, *m* represents the thermal softening exponent, and $T^{*}$ is defined as (*T* − *T*_*m*_)/(*T* − *T*_*r*_) with *T*_*m*_ is the material’s melting temperature and *T*_*r*_ is the reference temperature. ε represents the equivalent plastic strain. $\dot{\varepsilon }$ and ${\dot{\varepsilon }}_{0}$ represent the current and reference strain rates, respectively.

The Johnson-Cook parameters for the solid titanium matrix [28] are provided in Table 3.

**Table 3.** Johnson-Cook constitutive model parameters for solid titanium matrix.

Material	*A* (MPa)	*B* (MPa)	*n*	*m*	*C*
Ti	182.55	441.12	0.5343	1.394	0.343

Based on the established SHPB simulation model for titanium foam, impact simulation experiments were conducted on porous titanium with different relative densities. Additionally, a broad strain – rate range (3000–8000 s^−1^) and a temperature range (25–550 °C), as listed in Table 4, were chosen, aiming to investigate the influence of relative density on the dynamic response of porous titanium under high – temperature and high – strain – rate conditions.

**Table 4.** Matrix of strain rates and temperatures simulated in SHPB tests.

Temperature (℃)	25	250	400	550
Strain rate (s^−1^)	3000	3000	3000	3000
	6000	6000	6000	6000
	8000	8000	8000	8000

**Figure 8.**
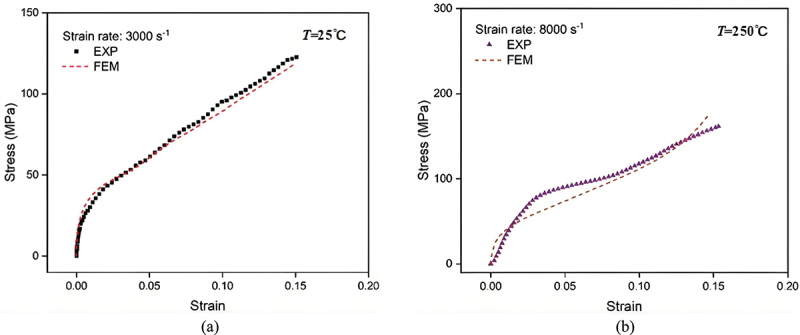
Comparison of simulated and experimental nominal stress-strain curves of porous titanium under different loading conditions: (a) 3000 s^−1^, 25°C and (b) 8000 s^−1^, 250°C. Reproduced with permission from ref [29], copyright © 2025 Taylor & Francis.

A comparison of the nominal stress-strain relationships of porous titanium under different loading conditions (with applied strain rates and temperatures of 3000 s^−1^, 25°C and 8000 s^−1^, 250°C, respectively) is presented in Figure 8, and the accuracy of the finite element model was evaluated by quantifying the discrepancy between the simulated and experimental flow stresses using Equation (7):

(7) $$\Delta =\left|\frac{\sigma_{\mathrm{EXP}}-\sigma_{\mathrm{FEM}}}{\sigma_{\mathrm{EXP}}}\right|\times 100\%$$

where *σ*_*EXP*_ and *σ*_*FEM*_ denote the flow stresses measured experimentally and obtained from simulations, respectively. The average error in predicted flow stress across the compared conditions was 14.8%. Furthermore, as shown in Figure 8, the experimental and simulated stress-strain curves exhibit a high degree of consistency up to a strain of approximately 0.1, demonstrating the model’s capability to capture the key features of the dynamic compression response of porous titanium. Moreover, consistent conclusions have also been reached in our previous work [29].

## 3. Results and discussion

### 3.1. Macroscopic dynamic stress-strain response

**Figure 9.**
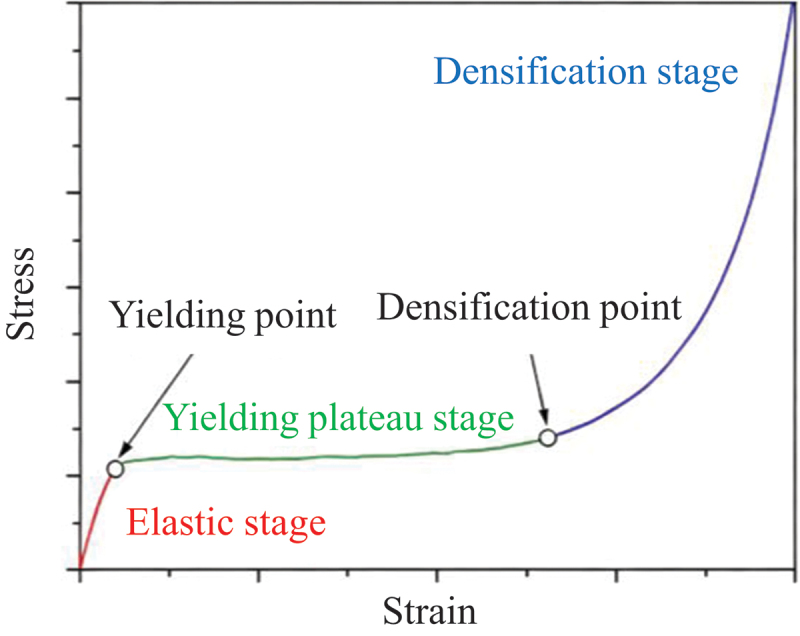
Schematic of the stress-strain curves under dynamic loading.

Figure 9 Schematic diagram illustrating the characteristic regimes of a porous material’s compressive stress-strain curve and the definition of key parameters. The dynamic compressive stress-strain response of porous titanium under impact loading typically exhibits three distinct regimes [30]: (i) linear elasticity, characterized by a rapid initial stress increase; (ii) plateau, marked by quasi-constant or slowly rising stress with significant strain accumulation; and (iii) densification, where stress rises sharply due to pore collapse and material compaction.

**Figure 10.**
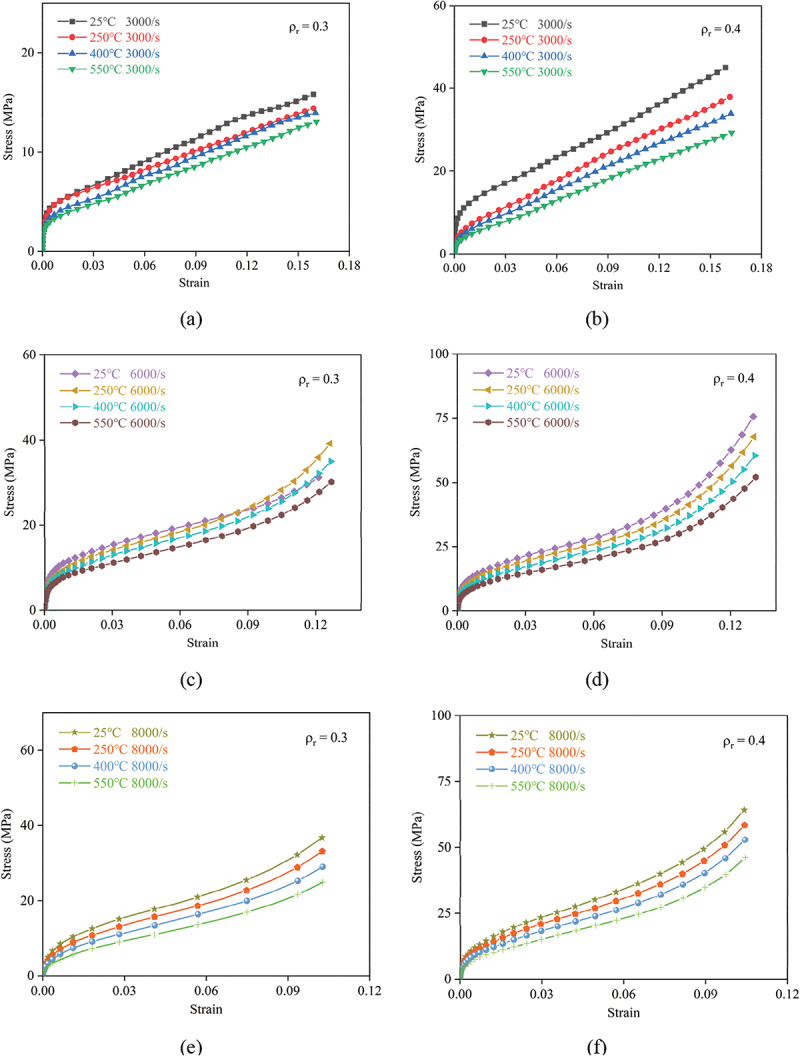
Dynamic compressive nominal stress-strain curves of porous titanium under SHPB loading for relative densities (a, c, e) *ρ*_*r*_ = 0.3 and (b, d, f) *ρ*_*r*_ = 0.4 at strain rates of 3000 s^−1^, 6000 s^−1^, and 8000 s^−1^.

**Figure 11.**
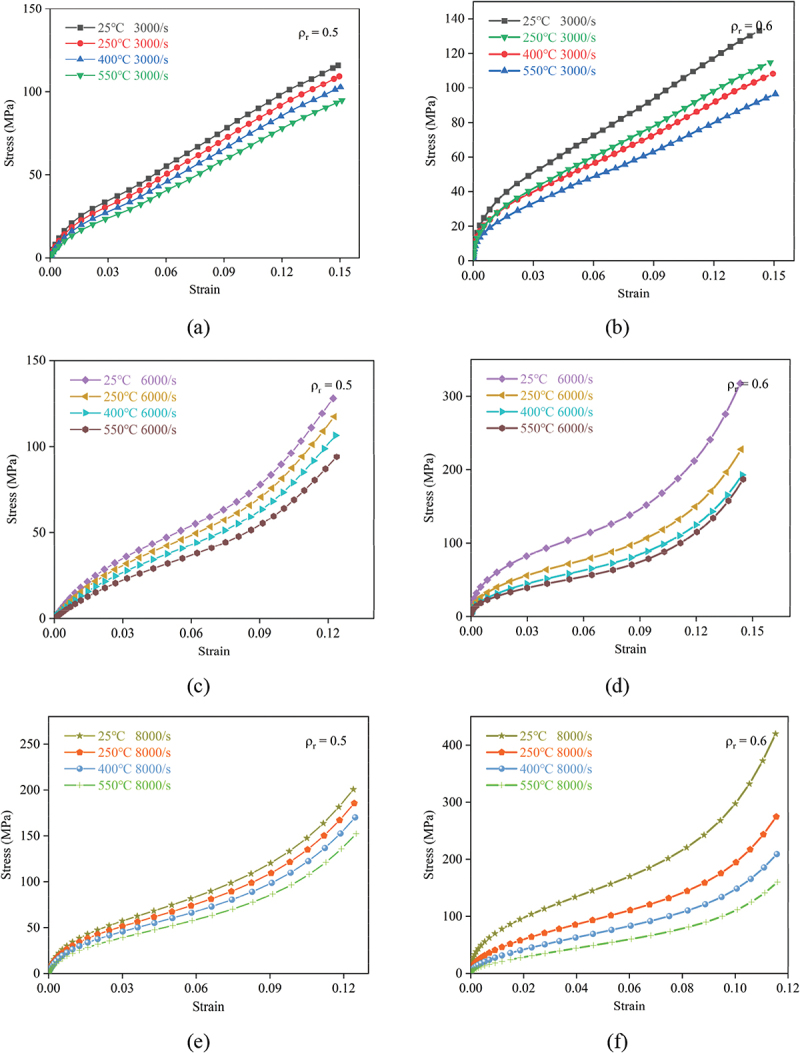
Dynamic compressive nominal stress-strain curves of porous titanium under SHPB loading for relative densities (a, c, e) *ρ*_*r*_ = 0.5 and (b, d, f) *ρ*_*r*_ = 0.6 at strain rates of 3000 s^−1^, 6000 s^−1^, and 8000 s^−1^.

As illustrated in Figures 10 and 11, the linear elastic, plateau, and densification phases in the stress-strain curves of porous titanium exhibit distinct evolutionary patterns, regulated by strain rate, temperature, and relative density, with the interplay of these factors dictating the overall mechanical response.

In the linear elastic regime, where stress rises sharply with strain, the extent of stress increment is inherently tied to relative density. For instance, at a strain rate of 3000 s^−1^ and 25°C, specimens with a relative density of 0.6 (Figure 11) exhibit a significantly steeper initial slope and higher stress values in the elastic range compared to those with 0.3 (Figure 10), underscoring that increased relative density enhances initial stiffness. This aligns with the observation that higher relative density generally yields elevated stress across the entire strain range under identical strain rate and temperature. Strain rate exerts a reinforcing effect: at 25°C and a relative density of 0.4, the elastic stress at 8000 s^−1^ surpasses that at 3000 s^−1^, manifesting the strain-rate strengthening effect. Conversely, temperature induces a softening influence at 6000 s^−1^ and 0.5 relative density, the elastic stress at 550°C is notably lower than at 25°C, reflecting thermal-induced reduction in yield stress at the onset of plastic deformation.

The plateau stage, characterized by quasi-linear strain hardening with attenuated stress increments, mirrors these parametric dependencies. Higher relative densities shift the plateau to higher stress levels; at 250°C and 6000 s^−1^, the plateau stress for 0.6 density (Figure 11) is roughly double that of 0.3 density (Figure 10), confirming that density governs the material’s flow stress during this phase. Elevated strain rates further enhance the plateau stress, as evidenced by the 8000 s^−1^ curves consistently lying above the 3000 s^−1^ counterparts under identical temperature and density conditions. In contrast, temperature elevation suppresses plateau stress; at 8000 s^−1^ and *ρ*_*r*_ = 0.4, the 550°C curve exhibits a lower plateau than the 25°C curve, which is attributed to temperature-induced softening that mitigates the material’s resistance to sustained deformation. Additionally, the plateau range expands with decreasing density; lower relative density (e.g. 0.3) allows for a broader strain interval before densification, as the abundant porosity provides more space for layer-by-layer collapse.

The initiation strain and stress magnitude of the densification phase, which is marked by a rapid stress surge due to pore compaction, are modulated by the three factors. Lower relative densities delay densification onset: under 25°C and 6000 s^−1^, 0.3 density specimens (Figure 10) enter densification at a higher strain than 0.6 ones (Figure 11), as more deformation is required to eliminate the larger initial porosity. Strain rate accelerates the stress rise in this phase; at 400°C and 0.5 density, the 8000 s^−1^ curve shows a steeper densification slope than 3000 s^−1^, amplifying the strain rate strengthening effect. Temperature, however, tempers the densification stress; the 550°C curves across all densities and strain rates exhibit a less pronounced stress surge compared to lower temperatures, as thermal softening facilitates pore collapse at reduced stress levels.

In summary, the results demonstrate that for a given relative density, both the yield stress (indicating the onset of the plateau regime) and the plateau stress exhibit strain-rate hardening (increase with strain rate) and thermal softening (decrease with temperature). Meanwhile, higher relative density consistently enhances stress values across all strain ranges under identical strain rate and temperature, underscoring its role as a pivotal determinant of mechanical performance. The synergistic interplay of relative density, strain rate, and temperature thus governs the evolutionary trajectories of the three phases, shaping the overall dynamic compressive response of porous titanium in SHPB tests.

### 3.2. Coupling effects of strain rate and temperature on yield stress

The yield stress of porous titanium demonstrates a well-known dependence on strain rate (increasing) and temperature (decreasing) under dynamic loading, consistent with the behavior of solid metals. However, under the coupled high-strain-rate and high-temperature conditions investigated here, their interaction becomes complex and competitive. For instance, with a relative density of 0.3, the yield stress at 6000 s^−1^ and 250°C is similar to that at 8000 s^−1^ and 400°C. This indicates that under dynamic loading, the individual effects are not merely additive; the thermal softening at elevated temperatures can significantly offset the strengthening achieved by high strain rates, a coupling effect whose magnitude is less pronounced in quasi-static loading. While each factor individually induces monotonic changes in yield stress (hardening with rate, softening with temperature), their coupling can lead to similar yield stress values under different combinations of strain rate and temperature. For instance, the yield stress at 6000 s^−1^ and 250°C closely approximates that at 8000 s^−1^ and 400°C. Similarly, for porous titanium with a relative density of 0.4, the yield stress at 3000 s^−1^ and 25°C is 9.92 MPa. Thermal elevation reduces this value to 5.19 MPa (−47.7%), 4.33 MPa (−56.4%), and 3.36 MPa (−66.1%) at 250°C, 400°C, and 550°C, respectively. At 6000 s^−1^, yield stresses reach 10.35 MPa, 9.24 MPa, 7.96 MPa, and 6.71 MPa, corresponding to deviations of −4.3%, +6.9%, +19.8%, and +32.4% relative to the 3000 s^−1^, 25°C baseline. Under 8000 s^−1^ loading, yield stresses further increase to 11.68 MPa, 9.40 MPa, 8.29 MPa, and 6.87 MPa, with coupled effects manifesting as −17.7%, +5.2%, +16.4%, and +30.7%, reinforcing the antagonistic relationship between strain-rate and temperature dependencies.

The dynamic yield stresses of porous titanium with relative densities of 0.5 and 0.6 are tabulated in Table 5. The coupling effect magnitude, expressed as the percentage deviation of yield stress from its value at 3000 s^−1^ and 25°C, quantifies the competition: a negative deviation indicates dominant strain-rate strengthening, while a positive deviation indicates dominant thermal softening. This metric provides a quantitative framework to assess the competitive interplay between strain-rate and temperature effects, enabling the identification of dominant deformation mechanisms under dynamic loading. Such insights are critical for tailoring porous titanium architectures to meet application-specific mechanical requirements, particularly in extreme environments involving elevated temperatures and high strain rates, such as aerospace, automotive, and defense systems for blast/ballistic resistance.

**Table 5.** Dynamic yield stress and magnitude of rate-temperature coupling effects for porous titanium with relative densities *ρ*_*r*_ = 0.5 and *ρ*_*r*_ = 0.6.

Strain rate (s^−1^)	*T*(℃)	Yield stress(*ρ*_*r*_ = 0.5)	The magnitude of rate-temperature coupling effects	Yield stress(*ρ*_*r*_ = 0.6)	The magnitude of rate-temperature coupling effects
3000	25	13.81	–	23.45	–
3000	250	11.14	19.3%	20.91	10.8%
3000	400	8.58	37.9%	16.34	30.3%
3000	550	6.9	50%	13.51	42.4%
6000	25	14.97	−8.4%	32.77	−39.7%
6000	250	12.82	7.2%	26.29	−12.1%
6000	400	10.83	21.5%	20.47	12.7%
6000	550	8.62	37.6%	16.34	30.3%
8000	25	26.24	−90.1%	49.19	−109.8%
8000	250	23.08	−67.1%	30.47	−29.9%
8000	400	19.61	−42%	22.67	3.32%
8000	550	16.15	−16.9%	18.9	19.4%

**Figure 12.**
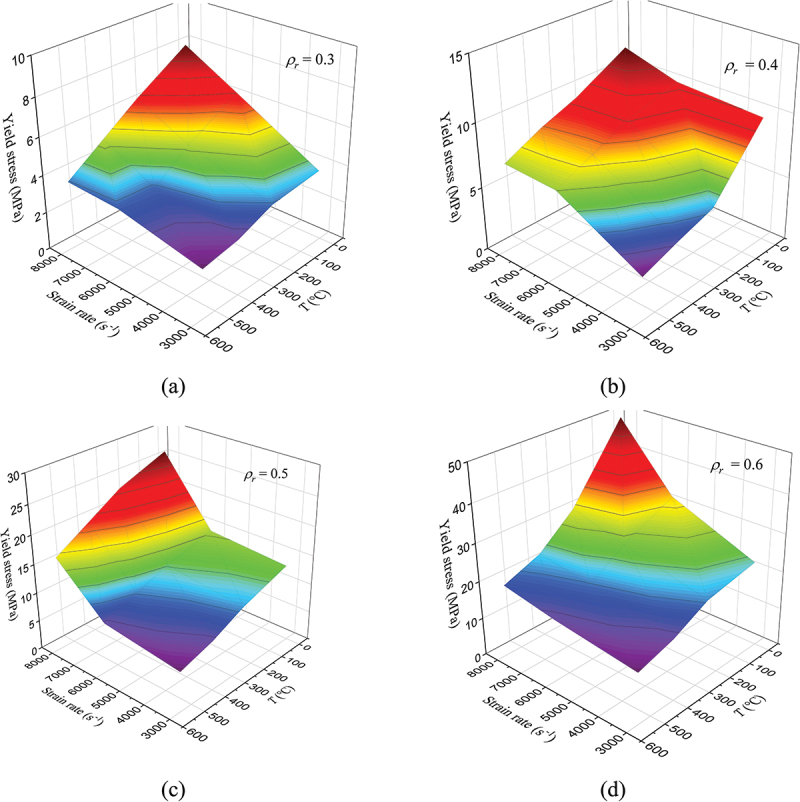
Dynamic yield stress of porous titanium as a function of relative density, strain rate and temperature.

With increasing relative density, the stress levels rise markedly, indicating a proportional enhancement in material strength. For example, at 8000 s^−1^ and 25°C, the yield stress of *ρ*_*r*_ = 0.6 porous titanium is 511.8% higher than that of *ρ*_*r*_ = 0.3. Corresponding increases at 250°C, 400°C, and 550°C are 382.9%, 355.2%, and 417.8%, respectively. As depicted in Figure 12, the yield stress across varying relative densities demonstrates monotonic increases with both relative density and strain rate but decreases with temperature. Furthermore, relative density significantly modulates the sensitivity to strain rate and temperature. Specimens with higher relative density exhibit more pronounced coupling effects. This amplification is attributed to the increased volume fraction of solid material, which enhances the contributions of both dislocation-mediated mechanisms (responsible for strain-rate hardening) and thermally activated processes (responsible for softening). This implies that high-density porous titanium, with its superior strength-to-weight ratio, holds unique advantages for applications demanding both lightweight properties and mechanical robustness.

The strong dependence of yield stress on relative density and the rate-temperature coupling effect provides a foundational guideline for designing porous titanium components for specific dynamic performance. For instance, a lower relative density may be selected for applications requiring a compliant response, while a higher relative density is essential for components demanding high specific strength under impact and elevated temperature conditions, such as in aerospace and automotive protective structures.

### 3.3. Localized deformation and surface densification

**Figure 13.**
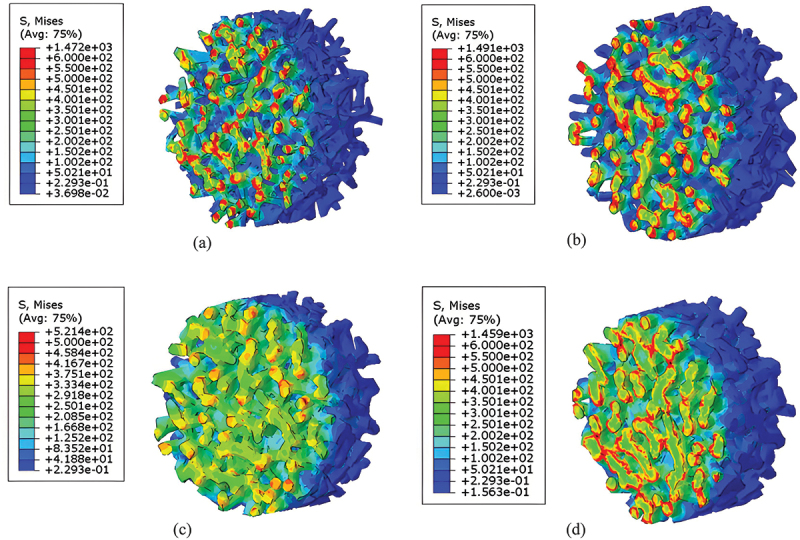
Contour plots of maximum principal stress (MPa) in porous titanium models under SHPB impact loading (3000 s^−1^, 250 °c) at time *t* = 0.322 ms for relative densities: (a) *ρ*_*r*_ = 0.3, (b) *ρ*_*r*_ = 0.4, (c) *ρ*_*r*_ = 0.5 and (d) *ρ*_*r*_ = 0.6.

**Figure 14.**
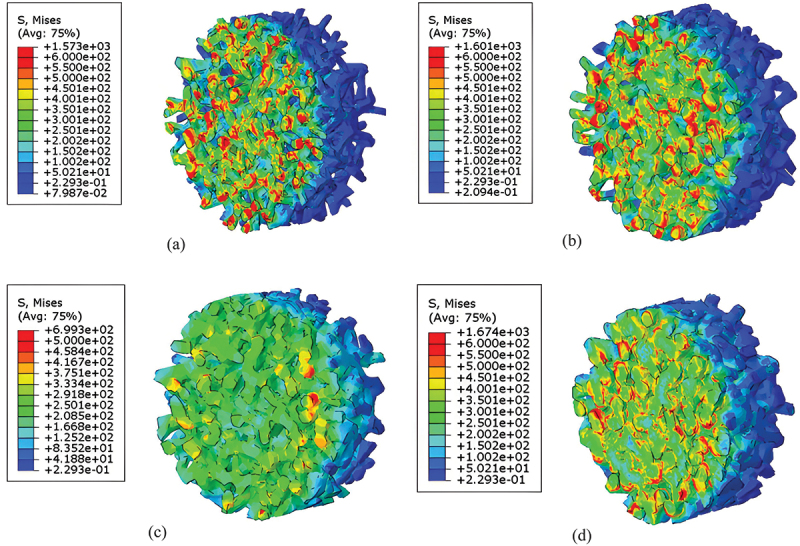
Contour plots of maximum principal stress (MPa) in porous titanium models under SHPB impact loading (3000 s^−1^, 250 °c) at time *t* = 0.350 ms for relative densities: (a) *ρ*_*r*_ = 0.3, (b) *ρ*_*r*_ = 0.4, (c) *ρ*_*r*_ = 0.5 and (d) *ρ*_*r*_ = 0.6.

Figures 13 and 14 present the contour maps of maximum principal stress in porous titanium specimens under dynamic loading. In these models, the solid lattice framework represents the cell walls (or struts), while the void spaces between them are the pores. The highest stress levels, indicated by red and yellow regions in the contour, define the stress concentrations. Notably, the peak stress within the solid material (cell walls) increases with relative density. Furthermore, stress concentrations are universally observed at the impact surface in all models, with localized high-stress zones predominantly distributed at the junctions where multiple struts meet (nodes) and the regions of strut bending.

Finite element simulations reveal dynamic stress redistribution in porous titanium, particularly the migration of stress concentration zones during progressive plastic deformation. Specifically, stress concentrations shift from regions undergoing active yielding to adjacent undeformed areas, a phenomenon observed corroborated in the contour maps. During this process, the majority of struts at the impacted surface deflect toward internal void spaces, while a minority exhibit outward buckling. This results in continuous deformation leading to localized densification near the impact surface. Upon reaching a critical densification threshold, the equivalent plastic stress begins to stabilize, accompanied by a deceleration in global cell wall deformation rates. As deformation progresses further, the stress concentrations near the impact surface gradually diminish, accompanied by a transition towards more homogeneous deformation throughout the specimen. This shift in behavior corresponds to the partial closure of pores, the collapse and compaction of cell structures, and a consequent reduction in local porosity, particularly in the impacted region.

**Figure 15.**
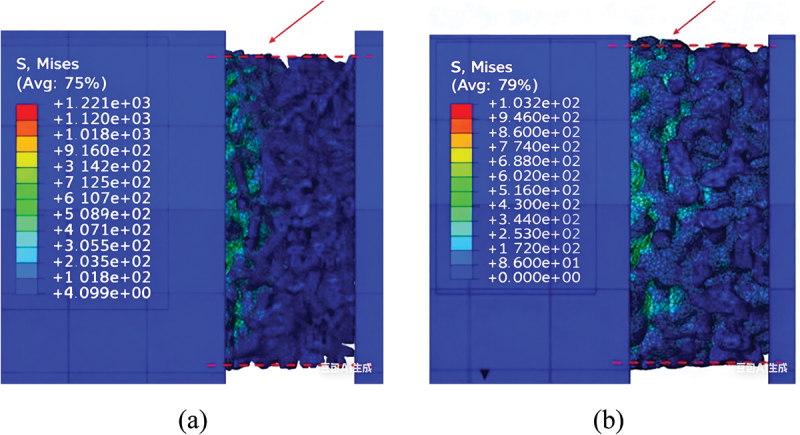
Finite element simulation results comparing the lateral deformation profiles (outlined in White for clarity) of porous titanium under dynamic uniaxial compression: (a) *ρ*_*r*_ = 0.3 exhibits minimal lateral expansion, consistent with layer-by-layer collapse. (b) *ρ*_*r*_ = 0.6 exhibits significant barreling, indicating bulk like triaxial deformation.

The finite element simulations reveal that the deformation modes of porous titanium under dynamic uniaxial compression exhibit significant dependency on relative density. Theoretically, materials with lower relative densities exhibit greater compressibility, whereas those approaching *ρ*_*r*_ = 1 demonstrate near-incompressibility akin to fully dense metals. Figure 15 illustrates the significant influence of relative density on lateral deformation under dynamic uniaxial compression. Simulation results for specimens with low relative density (*ρ*_*r*_ ≤0.4) exhibit minimal lateral expansion, a pattern consistent with a layer-by-layer collapse mechanism that primarily accommodates strain axially. In stark contrast, specimens with higher relative density (*ρ*_*r*_ = 0.6) demonstrate pronounced lateral expansion (barreling), indicative of a transition towards bulk-like, triaxial compressive deformation within the denser solid network.

Furthermore, distinct mesostructural deformation mechanisms govern porous titanium across varying relative densities, with a progressive transition observed around *ρ*_*r*_ ≈0.5:

In predominantly low relative density specimens (*ρ*_*r*_ < 0.5): In this regime, the slender struts (characterized by a high aspect ratio, i.e. length significantly greater than thickness) can be idealized as Euler beams, with deformation dominated by bending. Consequently, macroscopic strain hardening arises from sequential cell buckling and intercell interactions under compressive loading.

In predominantly high relative density specimens (*ρ*_*r*_ > 0.5): As the relative density increases, the struts become shorter and thicker, and the pore volume decreases significantly. The deformation transitions from strut bending to a state where the interconnected solid material experiences a complex three-dimensional, multiaxial stress state, dominated by triaxial compression. This shift leads to triaxial compressive deformation within the solid material, correlating with elevated yield stresses and pronounced strain hardening in macroscopic mechanical responses.

Collectively, increasing relative density amplifies lateral expansion during uniaxial compression while fundamentally altering the microscale deformation mechanism: with a notable shift from cell-wall buckling in low-density variants to matrix-dominated triaxial compression in high-density architectures.

**Figure 16.**
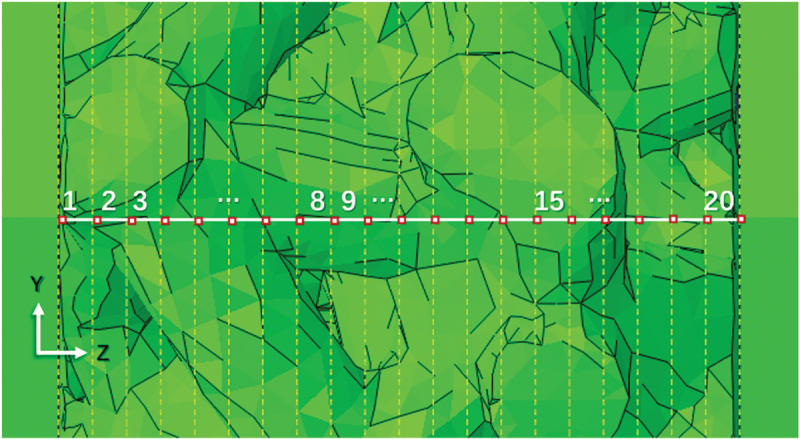
Schematic illustrating the division of the specimen model into 20 axial regions (SET1 SET20 from impact end to fixed end) for quantitative localized deformation analysis.

The deformation response of porous titanium under impact loading was investigated through regionally partitioned finite element analysis. To quantify localized deformation characteristics, the model was subdivided into 20 axial zones (denoted SET1 - SET20, from impact to constrained end, Figure 16). Node displacement data from Frame 44 (initial deformation) to Frame 64 (near-final deformation), sampled at single-frame intervals, were processed in MATLAB to compute averaged regional displacements.

**Figure 17.**
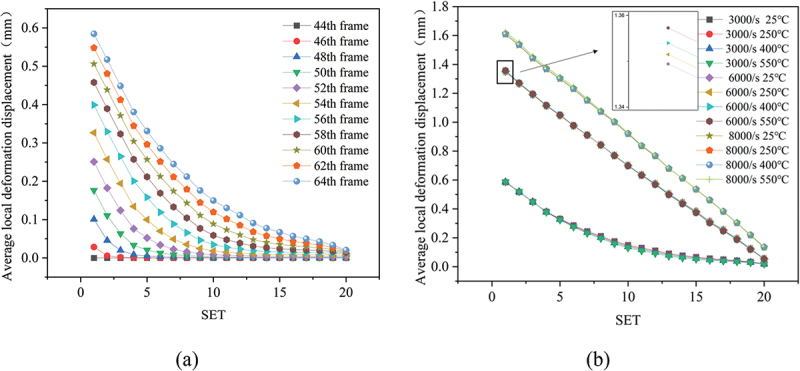
Localized deformation in porous titanium (*ρ*_*r*_ = 0.3): (a) evolution of average displacement per region over time under 3000 s^−1^, 25°C loading; (b) average displacement per region at Frame 64 across all tested strain rates and temperatures.

As illustrated in Figure 17a for a 0.3 relative density specimen (3000 s^−1^, 25 °C), deformation was predominantly localized within the first five zones (SET1-SET5) at the impacted surface, consistent with energy absorption patterns described in Section 3.2. Progressive deformation propagation into the specimen interior was observed with increasing frame counts. Figure 17b details Frame 64 displacement distributions under combined strain rate (3000–8000 s^−1^) and temperature (25–550 °C) variations. Maximum and minimum average displacements reached 1.62 mm (8000 s^−1^, 550 °C) and 0.58 mm (3000 s^−1^, 25 °C), respectively. Specimens at 6000 s^−1^ and 8000 s^−1^ exhibited quasi-linear displacement gradients, indicating uniform densification and stabilized plastic flow-a behavior aligned with the plateau-to-densification transition in stress-strain curves. Conversely, the 3000 s^−1^ profile followed a power-law decay, revealing incomplete stress wave propagation and persistent layer-by-layer collapse deformation.

Furthermore, the influence of temperature on the local displacement distribution (Figure 17b) is relatively minor compared to that of strain rate. Its primary effect manifests as a global shift in the stress-strain response (Section 3.1), rather than altering the localized deformation pattern. As shown in the magnified partial view of Figure 17b, elevated temperatures induce relatively higher deformation in porous titanium due to softening effects. However, the influence of temperature on the pattern of localized deformation is relatively minor compared to the dominant effect of strain rate. To isolate localized deformation patterns from rigid body motion and cumulative displacement, we introduce the relative local deformation displacement, defined as the difference in average displacement between adjacent regions.

**Figure 18.**
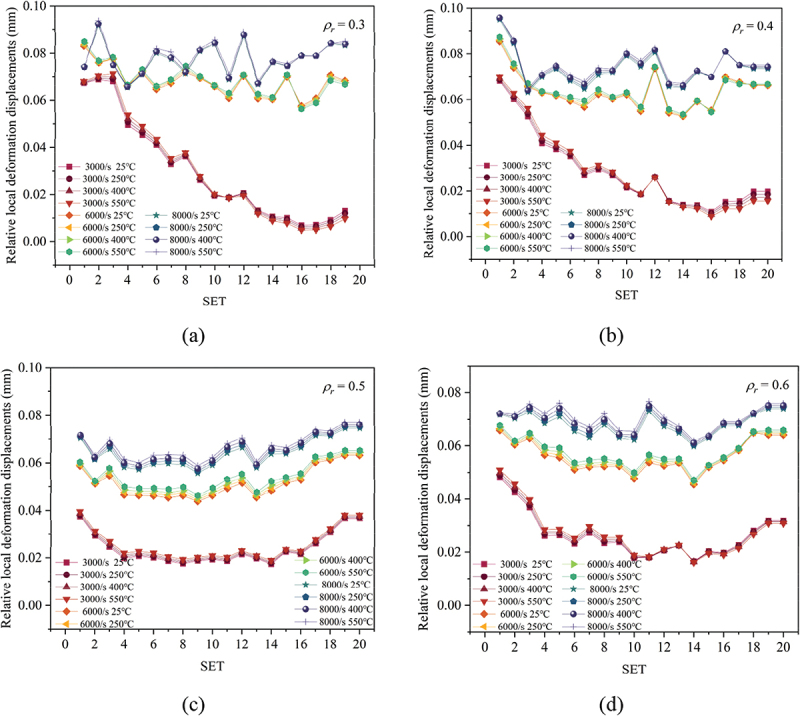
Average relative local deformation displacement per region at Frame 64 for porous titanium under different loading conditions: (a) *ρ*_*r*_ = 0.3, (b) *ρ*_*r*_ = 0.4, (c) *ρ*_*r*_ = 0.5 and (d) *ρ*_*r*_ = 0.6.

The localized deformation characteristics of porous titanium under dynamic loading are influenced by relative density, strain rate, and temperature. By calculating the average local displacement difference between each region and adjacent regions, the average relative deformation displacement of each region in porous titanium specimens with different relative densities was determined, as shown in Figure 18. Overall, porous titanium specimens with varying relative densities exhibit higher relative deformation magnitudes at an 8000 s^−1^ strain rate, with a monotonic decrease in relative deformation magnitudes at different positions as the strain rate decreases. At low strain rates (3000 s^−1^), low-*ρ*_*r*_ (0.3) specimens exhibit deformation concentrated at the impact end, whereas specimens with higher relative densities (e.g. *ρ*_*r*_ = 0.5–0.6) show a more uniform deformation distribution, similar to the relative deformation patterns observed at high strain rates, albeit with reduced amplitudes. Specifically, the maximum relative local deformation displacements for *ρ*_*r*_ = 0.3 and 0.4 specimens are 0.09 mm, whereas those for *ρ*_*r*_ = 0.5 and 0.6 specimens are 0.07 mm.

Combined with the deformation characteristics, the results indicate that lower relative density porous materials display more pronounced layer-by-layer collapse under dynamic deformation, accompanied by greater relative local deformations. This occurs because the deformation behavior progressively transitions with increasing relative density, and at high relative densities, it approximates that of fully dense metallic materials, whereas low relative density porous materials experience sequential energy transfer from the impacted end to the central and trailing regions during deformation. As the cell unit’s position and distance from the impact end increase, the relative local deformation displacement first decreases and then increases. This distribution arises due to two factors: (i) The cell walls near the specimen ends (impact and constrained) are often less constrained laterally and may have geometric imperfections introduced during model generation or physical manufacturing, rendering them more susceptible to buckling and deformation. (ii) The propagating stress wave initially activates and deforms the cell units in its path (near the impact end), while regions ahead of the wave front experience less deformation until the wave arrives.

Similarly, the influence of temperature on the local deformation characteristics of porous titanium is primarily manifested as an overall shift in the stress-strain curves. Elevated temperatures induce relatively higher deformation in porous titanium due to softening effects, though the overall magnitude remains limited. This is primarily because the dominant effect of elevated temperature is a global reduction in flow stress (thermal softening), which scales the overall displacement magnitude but does not fundamentally alter the underlying deformation mechanism or localization pattern governed by relative density and strain rate.

## 4. Conclusions

This article investigates the mechanical behavior of porous titanium with varying relative densities under dynamic uniaxial compression. Based on Split Hopkinson Pressure Bar (SHPB) simulation tests, dynamic impact simulations were conducted across a relative density range of 0.3–0.6, strain rates of 3000 s^−1^-8000 s^−1^, and temperatures of 25 °C-550°C. Key findings regarding the stress response, global deformation characteristics, and localized deformation features were analyzed quantitatively.

1. Relative density is the paramount factor governing the dynamic compressive response of porous titanium. Increasing the relative density from 0.3 to 0.6 results in a remarkable 511.8% increase in yield stress. Across the investigated range of relative densities (0.3–0.6), yield stress consistently increases with both relative density and applied strain rate, while it decreases with increasing temperature.
2. Strain rate significantly amplifies the magnitude of localized deformation, as quantified by the relative local deformation displacement. Under low strain rate (3000 s^−1^) loading, low-density specimens (*ρ*_*r*_ = 0.3) exhibit deformation concentrated near the impact end, characteristic of sequential layer collapse. In contrast, high-density specimens (*ρ*_*r*_ = 0.5–0.6) display a more uniform deformation distribution even at low rates. The maximum values of relative local deformation displacement were approximately 0.09 mm for low-density specimens and 0.07 mm for high-density specimens, reflecting the more pronounced localization associated with the layer collapse mechanism in low-density foams.
3. The dominant deformation mechanism exhibits a density-dependent transition. Analysis of lateral deformation patterns and localized strain suggests that a shift occurs around a relative density of *ρ*_*r*_ ≈0.5. At lower relative densities (*ρ*_*r*_ < 0.5), deformation is primarily governed by strut bending and sequential, layer-by-layer collapse. At higher relative densities (*ρ*_*r*_ > 0.5), the deformation is increasingly dominated by triaxial compression within the interconnected solid matrix. The material exhibits competitive coupling between strain-rate hardening and thermal softening. Notably, the magnitude of this rate-temperature coupling effect is amplified in specimens with higher relative density, attributable to the increased volume fraction of solid material participating in both dislocation-mediated hardening and thermally activated softening processes.

These findings offer practical guidance for designing porous titanium components. Lower relative densities are suited for applications demanding efficient energy absorption through layer-by-layer collapse while higher relative densities are optimal for structural parts requiring high specific strength under thermo-mechanical impact. The identified trends are constrained within the studied parameter ranges of relative density (0.3–0.6), strain rate (3000–8000 s^−1^), and temperature (25–550 °C). Their extrapolation beyond these bounds requires further investigation.

## Data Availability

The data that support the findings of this study are available from the corresponding authors upon reasonable request.
